# THE ASSOCIATION OF PERCEIVED SOCIAL SUPPORT AND SOCIAL ISOLATION WITH SUBJECTIVE COGNITIVE DECLINE IN OLDER ADULTS

**DOI:** 10.1093/geroni/igae098.0472

**Published:** 2024-12-31

**Authors:** Iftekhar Amin, Syeda Jesmin

**Affiliations:** University of North Texas at Dallas, Dallas, Texas, United States; University of North Texas at Dallas, Dallas, Texas, United States

## Abstract

**Objective:**

Early detection and diagnosis of subjective cognitive decline (SCD) has been prioritized in the Healthy People 2030 objectives. Two specific objectives are to increase the proportion of adults with SCD who know they have it and who have discussed this with a care provider. Although the literature shows that women may have faster cognitive decline than men, research is lacking on whether differential levels of social support and isolation play any role in the prevention and delay of the onset of cognitive decline in older men and women.

**Methods:**

We used data from the 2022 Behavioral Risk Factor Surveillance System (BRFSS, N= 4,45,132). Logistic regression models have been used to examine the association between social support, isolation, and SCD in a sample of community-dwelling older adults with SCD aged over 65.

**Results:**

Overall, among 31,625 respondents over age 65, nearly 10.1% reported experience of confusion or memory loss. Lower scores on the composite measure of social support and isolation were associated with greater reports of SCD for both men (OR 2.6, 95% CI = 2.3, 2.9) and women (OR 2.9, 95% CI = 2.6, 3.2). Compared to Hispanic older adults, the effects of isolation and lack of social support on the odds of SCD were higher for Whites (OR 2.0, 95% CI = 1.8, 2.3), and Blacks (OR 1.4, 95% CI = 1.2, 1.7).

**Conclusion:**

Our findings highlight the importance of addressing social support and social connectedness in cognitive decline-related health promotion efforts for older adults, women in particular.

